# The more favorable attitude of the citizens toward GMOs supports a new regulatory framework in the European Union

**DOI:** 10.1080/21645698.2020.1795525

**Published:** 2020-08-12

**Authors:** Mihael Cristin Ichim

**Affiliations:** “Stejarul” Research Centre for Biological Sciences, National Institute of Research and Development for Biological Sciences, Piatra Neamt, Romania

**Keywords:** Transgenic plants, genetically modified crops, confined field trial, environmental release, commercial cultivation, GM food, Eurobarometer, citizen attitude

## Abstract

Since 1996 till 2018, the global area cultivated with GM crops has increased 113-fold, making biotech crops one of the fastest adopted crop technology in the past decades. In the European Union, only two countries still cultivate one available transgenic crop event on minor hectarage. Moreover, the number of notifications for confined field trials has dramatically dropped in the last decade. All these are happening while the EU legislation on GM crops has come under severe criticism. The percentage of EU citizens concerned about the presence of GMOs in the environment has decreased from 30% (in 2002) to 19% (in 2011), while the level of concern about the use of GM ingredients in food or drinks has decreased from 63% (in 2005) to 27% (in 2019). The steadily increasing acceptance of the EU citizens of GMOs in the environment and food, as it was recorded by Eurobarometers, should additionally ease the way and support a positive change of the legal framework that regulates the GM crops’ testing and commercial cultivation in the EU.

## Introduction

At the global level, between 1992 and 2018, 4,349 approvals have been granted by regulatory authorities from 70 countries (42 + EU-28) for food (2,063), feed (1,461), and environmental release or commercial cultivation (825) of genetically modified (GM) plants. Only 202 approvals have been granted in the European Union (EU): for food (99), feed (100), and cultivation purposes (3 maize events with insect resistance or glufosinate herbicide tolerance).^1^ In 2018, 26 countries grew 191.7 million hectares worldwide with GM crops, in the 23^rd^ year of their global commercialization. At the global level, the USA, Brazil, Argentina, Canada, and India remain the top five GM crops-growing countries, each of them having a close to 100% adoption rate. Since 1996 till 2018, the global area cultivated with GM crops has increased 113-fold, accumulating 2.5 billion hectares in this period, making biotech crops one of the fastest adopted crop technology in the past decades.^1^ In the EU, the commercially cultivated area with GM crops continues to remain minor through 2018. In each of the last 8 years, the total area commercially cultivated with GM crops in the EU was above 100,000 ha but fluctuates yearly, without ever reaching 150,000 ha during the uninterrupted 21-year long history of GM crops commercial cultivation in Europe.^1–3^ All these happening while the EU legislation on GM crops has come under severe criticism, especially in the recent years.^4,5^ It was argued that the European regulatory framework for GMOs does not satisfy the criteria of legal certainty, nondiscrimination, and scientific adaptability.^6^ The urgent need for a new, evidence-based, flexible, and proportionate GMO regulatory framework has been scientifically argued,^7^ but also politically demanded for.^8^ The long-awaited reform should focus on traits, not processes, which will stimulate innovation and public debate^5^ and allow to the EU to start to capitalize on the plant breeding opportunities afforded by the New Plant Breeding Techniques (NPBTs).^9^

## Confined Field Trials with GM Plants in the EU

Most regulations worldwide stipulate that a new GM plant event has to be compared to its closest non-GM counterpart as an essential part of the risk assessment before market approval. The GM crop and its direct comparator, the non-GM (near-isogenic) variety with a history of safe use in the EU, should be grown in small-scale field trials. The confined field trials have to provide comparative data for the possible occurrence of differences, caused by intended and unintended effects, in composition, as well as in agronomic, phenotypic, and molecular characteristics of the two crop varieties.^10^

In the EU, before undertaking a confined field trial with a GMO, a notification must be submitted to the competent authority of the EU Member State (MS) within whose territory the environmental release is to take place. A summary of each notification received (summary notification information format – SNIF) is then sent to the European Commission (EC). The EU SNIF database^11^ continues to provide an accurate overview of all notifications circulated among the Member States since the implementation date (21 October 1991) of Directive 90/220/EEC.^12^

**Figure 1.**
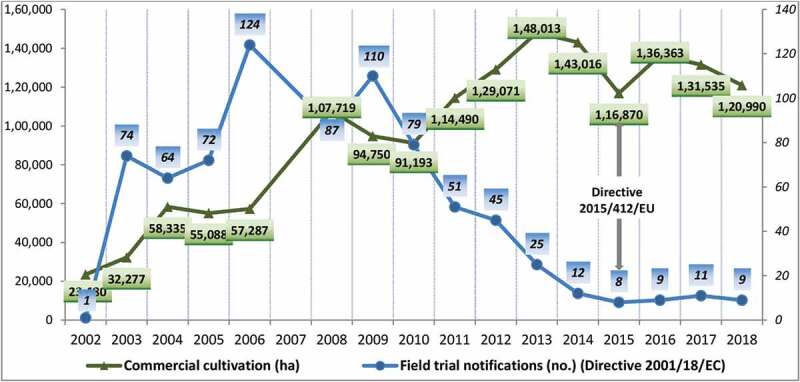
Evolution of the GM plant field trial notifications and commercial cultivation of GM crops in EU (2002–2018).

The EU SNIF database (version February 2002) (https://gmoinfo.jrc.ec.europa.eu/overview-main.aspx) for GMO field trial notifications^12^ was previously analyzed and it was found that during the first decade (1991–2001), a total of 1,687 field trial notifications were recorded for GM plants. However, during the years encompassing the *de facto* moratorium (1998–2001) the number of notifications dropped precipitously by 76%.^13^ Soon after, the Directive 90/220/EEC was amended by the Directive 18/2001/EC^14^ and the Joint Research Center (JRC) of the EC continues to maintain the GMO register where all the SNIFs submitted to the MS Competent Authorities under Directive 2001/18/EC (after 17 October 2002) are listed. Therefore, the register contains information about all the notifications for deliberate release into the environment (small-scale field trial) of GM plants for any other purposes than placing on the market (experimental releases) according to the provisions of the Directive 2001/18/EC (Part B) (https://gmoinfo.jrc.ec.europa.eu/gmp_browse.aspx). Since the implementation date of Directive 2001/18/EC till 2018, 868 notifications (the ones withdrawn were excluded) were registered in the EU SNIF database, a little over half (51%) of the notifications received in the previous decade under the Directive 90/220/EEC (Figure 1).

In the last 8 years, the number of notifications for confined field trials under Directive 2001/18/EC has dramatically dropped by 89%. In each of the last 5 years, only 10 (±2) new notifications were recorded in the EU SNIF database.

## Commercial Cultivation of GM Crops in the EU

While GM crops are commercially grown in 26 countries across all continents, in Europe, only two EU countries cultivated 120,990 hectares in 2018 (Figure 1), distributed among Spain (95%) and Portugal (5%).^1,15^ The EU countries cumulatively cultivated 1,736,725 hectares with GM crops in all 21 years (1998–2018),^1,3^ an insignificant 0.07% of the 2.5 billion hectares cultivated in total around the globe since 1996. The number of cultivating EU countries has also diminished in the recent years, as the farmers from Romania (in 2015), Czech Republic, and Slovakia (in 2017), have voluntarily stopped the commercial cultivation of GM crops.^3^ For farmers, the economic advantage offered by the adopted GM crops has to translate into profitability.^16^ In Europe, the incentives for commercial cultivation of the only GM plant event available, an insect-resistant (IR) maize variety designed to confer protection against certain lepidopteran insect pests,^17^ are decreasing considering the specific conditions offered by the market (*e.g*. agricultural commodity prices), agronomic conditions (*e.g*. pest pressure),^3^ or discouraging administrative regulations.^18^ Although the provisions of the EU GMO opt-out Directive 2015/412^19^ have allowed to a significant number of EU MSs to officially restrict the cultivation of genetically modified organisms (GMOs) in their territory, it did not change the *status quo* of the EU-28 countries as eleven countries have officially chosen to legally allow the commercial cultivation of GM crops.^3^

## The EU Citizens’ Attitude Toward GMOs and Their Use

### Monitoring the Public Opinion in the European Union

Since the early 1970 s, the European Commission (EC) monitors the public opinion in the EU member and candidate countries through the Eurobarometer program, a cross-temporal and cross-national comparative program of regularly repeated cross-sectional surveys.^20^ In addition, special Eurobarometers are used to monitor if citizens want the EU to act on a specific policy topic or support the Commission’s solution to a policy problem.^21^ All the Eurobatometers are made publicly available without restrictions^22^ and they were systematically searched for questions referring to genetically modified organisms, irrespective of their intended use or the year they were carried out or published.

### EU Citizens’ Attitude Toward GMOs

Back in 1999, “things being genetically modified” ranked 9^th^ (with 18.1%) among the eleven chief concerns of the EU-15 citizens but was the one that generated most divergent opinions across different countries. Moreover, the “use of genetically modified organisms, like genetically modified corn in other food products” ranked fifth out of nine overall concerns that worried the European citizens in 1999 more than they did five years before (in 1994).^23^

For two decades (1991–2010), the EC has assessed periodically, through Eurobarometer surveys about life sciences, the public attitude about GM food in Europe.^24^ The findings of the last survey from this series have shown a declining support among the EU countries by almost half, as the percentages of respondents who expressed their support for the development of GM food have steadily decreased from 50% (EU-15, 1996), 39% (EU-15, 1999), 42% (EU-15, 2002), 32% (EU-25, 2005), to 26% (EU-27, 2010).^24^ The same survey has recorded, for the first time, the opinion of the EU citizens about cisgenics, GM crops produced by adding only genes from the same, or closely related species that are crossable by conventional breeding. The hypothetical example of the cisgenic production of apples receives higher support (55%) than GM apples (33%), with the former attracting majority support in 24 countries.^25^ Cisgenics might have been seen as an example of the so-called second generation of GM crops, and the EU-27 respondents felt less uneasy about these GM apples, as they seem more natural, less problematic with regard to the environment, safer and more useful/promising.^24^

### The Use of Genetically Modified Organisms in Farming

**Figure 2.**
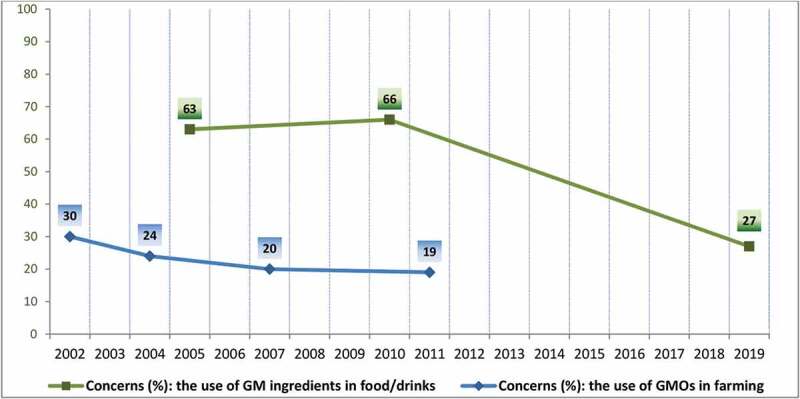
Evolution of the concerns expressed by the EU citizens toward the use of GM ingredients in food/drinks and the use of GMOs in farming (2002–2019).

The attitudes of Europeans toward the environment are also periodically surveyed through a special series of Eurobarometers. When for the first time, in 2002, the concerns about 25 different environmental issues were measured, the “use of GMOs” was ranked 18^th^, representing 30% from the worries expressed by the EU-15 citizens.^26^ Since then, a constant decreasing percentage of EU citizens has expressed their worries about the use of GMOs in farming: 24% (EU-25, 2004)^27^, 20% (EU-27, 2007)^28^, and 19% (EU-27, 2011)^29^ (Figure 2).

### The Use of Genetically Modified Ingredients in Food or Drinks

In 2005, when the first periodic Eurobarometer survey was initiated by the European Food Safety Authority (EFSA), 63% of the EU-25 citizens were worried about the use of genetically modified ingredients in food or drinks, ranking seventh among a total of 14 risks associated with food.^30^ Five years later, in 2010, 66% of the EU-27 citizens have indicated that same risk, *i.e*. the use if GM ingredients, as the sixth from 17 possible specific food-related risks surveyed.^31^ But, nine years later, only 27% of the EU-28 citizens were concerned about the presence of GM ingredients in food or drinks, eighth out of 15 food safety concerns surveyed in 2019.^32^ The level of concern of the EU consumers over this potential risk for food has decreased significantly, by more than half, over the surveyed period (2005–2019) (Figure 2).

## Discussion

The total number of notifications for confined field trials with GM plants in the last 28 years (1991–2018) in the EU is 2,555, as recorded in the EU SNIF database: 1,687 under Directive 90/220/EEC (1991–2001) and 868 under Directive 18/2001/EC (2002–2018). In the last 4–5 years of each of the regulatory Directive, a significant decline of notification was registered. The number of notifications received in the 17 years under Directive 18/2001/EC, is smaller in comparison with the number received in 10 years under the former Directive 90/220/EEC. Unfortunately, along with other negatively influencing factors, such as the increased hostility of radical farmers or “green” activists and the increasing number of vandalisms acts against the field trials with GM plants,^33,34^ the amendments brought by Directive 18/2001/EC have failed to improve and more efficiently regulate and support the testing in confined field trials as a necessary preliminary step in the authorization process as an approved GM event for commercial cultivation in the EU. The yearly evaluation of the notifications received during the 17-year period reveals the decline in field trial notifications in Europe which started in 2010 and continues till today. As of 25 July 2018, the date of the decision of the Court of Justice of the European Union (CJEU) that plants obtained with the new genome editing techniques must go through the same approval process as GM plants,^35,36^ the EU SNIF database contains also all the notifications for confined field trials with genome-edited plants. Many of the notification for field trials with GM plants have been done for scientific research purposes only, with no intentions to pursue an authorization for commercial cultivation in the EU but, considering that only three GM plants have been authorized for cultivation in the EU over the years, it means that only 0.12% from all notifications for confined field trials have finally resulted in the commercial cultivation of that particular GM plant in the EU. The European cultivated area represents in 2018 a negligible percentage (0.06%) from the one cultivated globally and it looks more like a small-scale (120,990 ha) and confined (to only two countries) field trial rather than a for-profit commercial cultivation on an entire continent. Only one GM plant event, initially approved back in 1998, is available in the EU and this represents an additional constraint toward a wider adoption of GM crops for commercial cultivation on the continent.

In contrast with the supportive opinions of the EU experts toward the use of GMOs,^37^ the attitude of the majority of the European citizens has long been suggested to remain a reluctant and skeptical one.^18,34,38-40^ In EU, citizens’ views of a variety of topics are regularly surveyed by the Eurobarometer studies commissioned by the European Commission to monitor the evolution of public opinion over several years, using uniform questionnaires and large numbers of interviews to obtain representative data.^18^ A different series of special Eurobarometers have recorded the evolution of the concerns expressed regarding the use of GMOs for farming or as food ingredients, and the percentage of EU citizens concerned about the use of GMOs has substantially decreased over the last two decades. This officially recorded trend supports, and also argues for, an immediate change of the present, not-fit-for the purpose, GMO regulatory framework to meet EU citizens’ expected benefits which GM crops could offer.

## Concluding Remarks

While the number of GM crops, authorized events, cultivating countries, and total area occupied with GM crops continues to steadily increase at a global level, in the whole of Europe there are only two countries left, commercially cultivating one GM event, on a small number of hectares. In direct relation with the modest cultivated area in the EU, a minor number of notifications for deliberate releases into the environment of genetically modified plants under Directive 2001/18/EC are officially registered in the recent years. In this context and in contradiction with the general belief that the Europeans are against GMOs, the concerns expressed by the EU citizens toward the use of GMOs in farming and food have significantly decreased in the past 20 years as recorded by the official Eurobarometer surveys commissioned by the European Commission. The more favorable attitude of the EU citizens toward GMOs should ease the way and encourage a positive change of the legal framework that regulates the GM crops’ testing and commercial cultivation in the EU.

## Data Availability

Data sharing is not applicable to this article as no new data were created or analyzed in this study.
